# Genetic Risk of Talon Cusp: Talon Cusp in Five Siblings

**DOI:** 10.1155/2019/3080769

**Published:** 2019-08-27

**Authors:** Nuha Abdel-Rahman Elmubarak

**Affiliations:** Department of Restorative Dentistry, National Ribat University, Khartoum, Sudan

## Abstract

Talon cusp is a rare dental anomaly that appears as a cusp-like projection on anterior teeth. Although numerous articles considering this anomaly have been published, this report has displayed a unique presentation of talon cusp. This case series is the first report in literature on which talon cusp has been presented in multiple siblings which highlights the genetic/familial component of the etiology. The report has also displayed unfamiliar morphological appearance (heart shape) of the talon cusp. Furthermore, talon cusp has shown an association with taurodontism in this report. Taurodontism has never been mentioned in the previous literature among the odontogenic variations that may associate talon cusp. A 25-year-old male has presented with talon cusps on the palatal surface of anterior teeth. Family history revealed four of his siblings had the same anomaly on anterior teeth. Talon cusps in the five cases cause clinical problems like occlusal interference, displacement and proclination of the anterior tooth, caries in the grooves delineating talon cusp, or pulp necrosis. Free dental treatment has been offered in the university clinic. However, they live in remote rural areas making it difficult to follow up with treatment.

## 1. Introduction

Talon cusp is a developmental dental anomaly in which an accessory cusp arises from the cingulum or cementoenamel junction of the anterior teeth in maxilla or mandible. It can affect deciduous or permanent teeth [1]. As all other cusps, talon cusp is composed of normal enamel underlined by dentin. It may or may not contain pulpal tissue [2]. However, large talon cusps, particularly those which stand away from the crown surface, more probably have pulp tissue [3].

Talon cusp was first reported in 1892 by Mitchell, who reported a maxillary central incisor with a horn-like bulge extending from the palatal surface [4]. It resembles an eagle's talon; hence, Mellor and Ripa give it the name talon cusp [5].

The etiology of talon cusp is not totally clear. Talon cusp arises during the morphodifferentiation stage of tooth development. It is proposed to be a normal consequence of outward bulging of the inner enamel epithelial cells (precursors of ameloblasts) and focal hyperplasia of the mesenchymal peripheral cells of dental papilla (precursors of odontoblasts) [2].

Clinically, some talon cusps possess sharp tips whereas others have smooth rounded tips. The tip of the cusp may stand away from the rest of the crown, or it may be fused with the tooth surface. Morphologically, talon cusps can be presented as horn-like, pyramidal, or conical shape [5–7].

On radiograph, talon cusp appears as a V-shaped radiopaque structure overlapping the affected crown with its apex directed incisally. Talon cusp on an unerupted tooth may be misinterpreted as mesiodens or compound odontoma. This misdiagnosis may lead to unnecessary surgical intervention in an attempt to remove it [2].

Based on histopathology, Danker et al. [8] and Uyeno and Lugo [9] described talon cusp as a form of dens evaginatus. Also, a previous literature review has considered talon cusp as a descriptive term of dens evaginatus on the lingual surface of anterior teeth [10]. However, depending upon the site, rate of incidence, and clinical appearance, dens evaginatus and talon cusp should be treated as separate anomalies [11]. The term dens evaginatus should be used for tubercles presented on the occlusal surfaces of premolars and molars [12].

Talon cusp usually occurs as an isolated anomaly rather than being part of any disorder [13]. Nevertheless, the anomaly has been reported in patients with Mohr syndrome [14], Rubinstein-Taybi syndrome [15], Sturge-Weber syndrome [7], and Ellis-van Creveld syndrome [16].

Talon cusp is not an innocent anomaly as large talon cusp may lead to many clinical problems including traumatic occlusion; displacement of the affected and opposing teeth; compromised esthetics in cases of facial talon cusp; plaque retention and caries susceptibility in the developmental grooves that delineate the cusp; attrition of the opposing teeth; periodontal problems; hypersensitivity, pulpal necrosis, and periapical pathosis due to excessive attrition; accidental cusp fracture; irritation of the tongue during speech and mastication; interference with tongue space; speech disturbance; breast-feeding problems; and temporomandibular joint pain due to excessive occlusal forces [4, 17–19].

The present report describes five siblings with talon cusps in all upper anterior teeth.

## 2. Case Presentation

A 25-year-old Sudanese male came to Students' Dental Clinic in the Faculty of Dentistry, National Ribat University, Sudan, to treat his lower molar. During oral examination, large tubercles have been noticed in the palatal surface of all upper anterior teeth and diagnosed as talon cusps. On family history, he mentioned that four of his siblings had the same tubercles on anterior teeth. The siblings have been contacted and invited for dental examination.

### 2.1. Case 1

**Figure 1:**
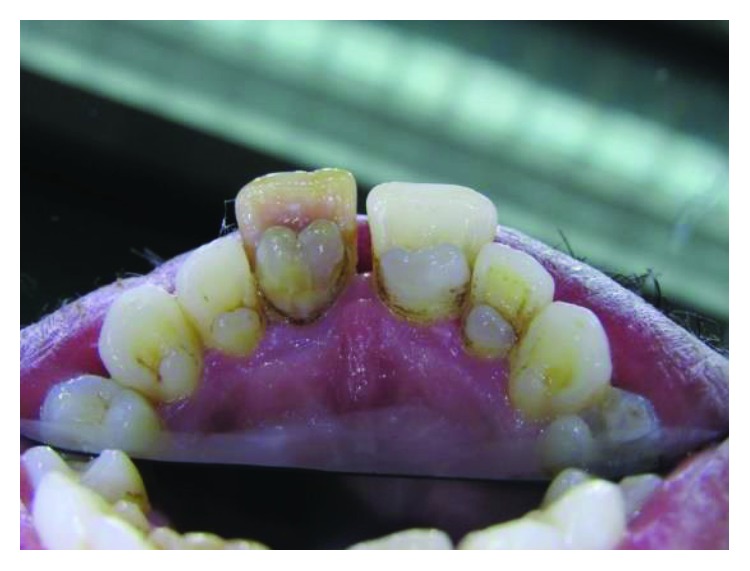
Case 1: heart-shape tubercles on both central incisors and typical talon-shape tubercles on both laterals and canines.

A 25-year-old male presented with talon cusps in all upper anterior teeth. Despite the name, talon cusps in both central incisors presented as heart-shaped tubercles rather than talon shape. However, lateral incisors and canines have the typical talon shape appearance (Figure 1).

Deep carious grooves separate the heart-shaped tubercles in both central incisors from the palatal surfaces of the teeth. Meanwhile, in the lateral incisors and canines, the grooves are not deep and not carious and the tubercles are in close proximity to the palatal surfaces. However, grooves in the lateral incisors are darkly stained.

**Figure 2:**
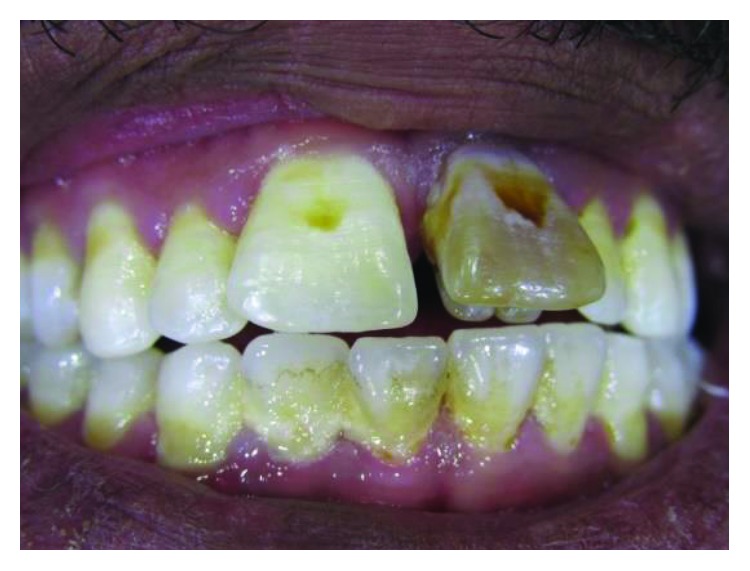
Case 1: discolored necrotic left central incisor (facial aspect).

All anterior teeth except the left central incisor have responded normally to the sensibility test. The left central incisor has no response to the sensibility test and shows discoloration (Figure 2).

**Figure 3:**
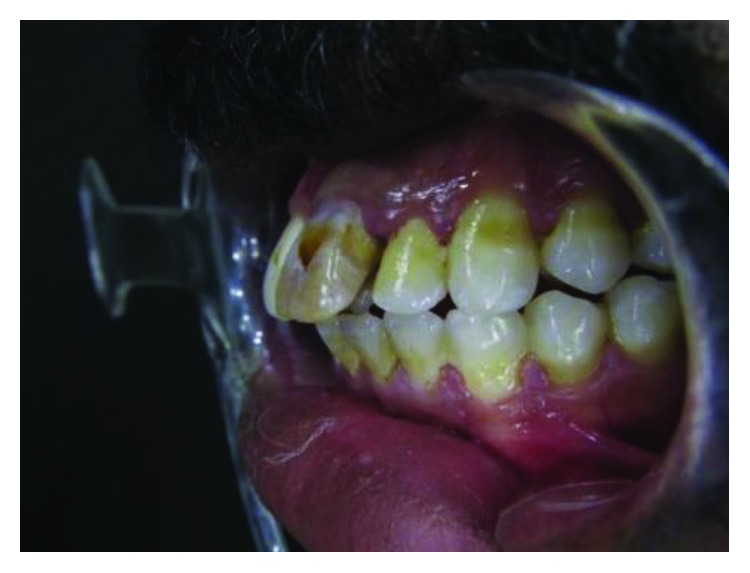
Case 1: proclination of upper teeth due to occlusal interference of the tubercles.

**Figure 4:**
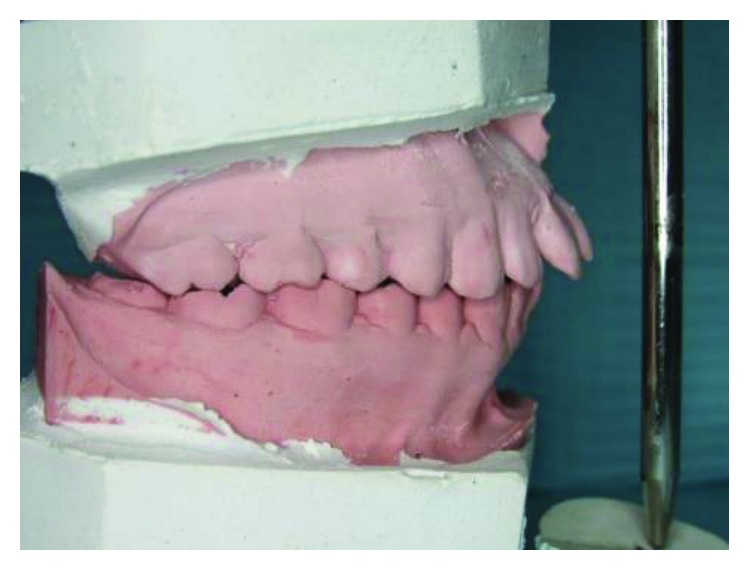
Case 1: study cast shows the large overjet of both central incisors.

The tubercles have been measured mesiodistally (MD) and incisogingivally (IG), and accordingly, talon cusps have been classified following Hattab et al.'s classification [2] (Table 1). Proclination of anterior teeth in this case occurs due to occlusal interference of the tubercles with the lower teeth. This proclination makes teeth at risk of trauma (Figures 2–4).

**Figure 5:**
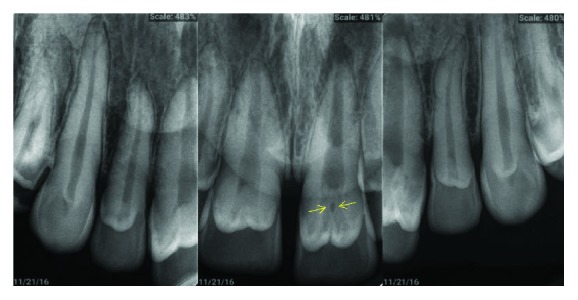
Case 1: periapical radiograph shows talon cusps in all upper anterior teeth. Notice the small radiolucent teardrop in the centre of the crown of the left central incisor (dens invaginatus).

**Figure 6:**
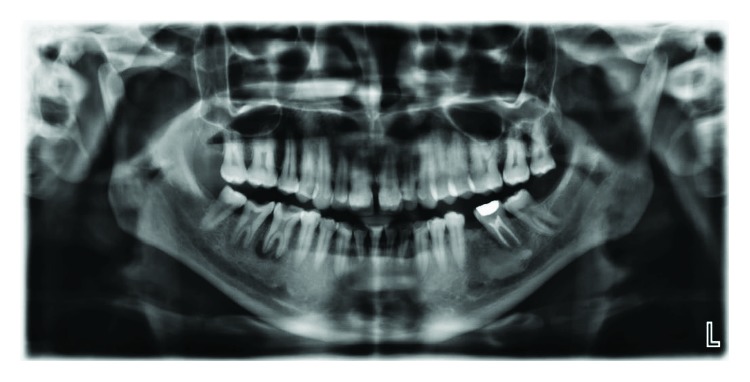
Case 1: dental panoramic tomography (DPT) shows taurodontism in molars.

Dental variations like dens invaginatus in the upper left central incisor (Figure 5) and taurodontism in posterior molars (Figure 6) are associated with talon cusps.

On radiographic examination for the left central incisor, radiolucency around its open apex has been noticed. It has been diagnosed as chronic apical periodontitis. Pulp pathology on the left central incisor may be due to one of two reasons.

1. *Trauma*. As shown in Figures 3 and 4, tubercle interference with lower teeth leads to proclination of upper incisors and makes them at risk to trauma. Proclination of the left central incisor is greater than other incisors, and this make it at a higher risk to trauma leading to pulp necrosis. Measurements of the tubercles revealed that the larger tubercle is located on the left central incisor, and this justifies why the left central incisor is more proclined than other anterior teeth.
2. *Dens Invaginatus*. Radiograph shows dens invaginatus on the left central incisor. Risk of pulp necrosis is well known in teeth with dens invaginatus if early precaution is not considered.

### 2.2. Case 2

A 21-year-old male, brother of case 1, also has talon cusps in the palatal aspect of all upper anterior teeth. Measurements of the tubercles have been presented in Table 1.

**Figure 7:**
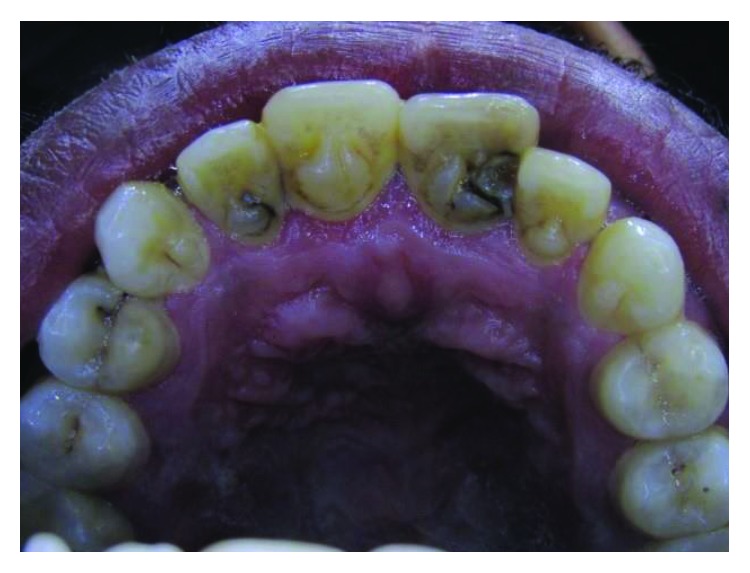
Case 2: talon cusps in all upper anterior teeth.

The right central incisor has been destructed by caries distally. So, MD dimension could not be measured. Talon cusps in both canines appear in a form of large bifid cingula (Figure 7).

According to Hattab et al.'s classification [2], both central incisors have been classified as Type 1 (talon), lateral incisors regarded as Type II (semitalon), and canines regarded as Type III (trace talon) (Table 1).

The sensibility test has revealed that all anterior teeth are vital except the right central incisor which is nonvital and discolored and the distal half of its tubercle destructed by caries (Figure 7).

**Figure 8:**
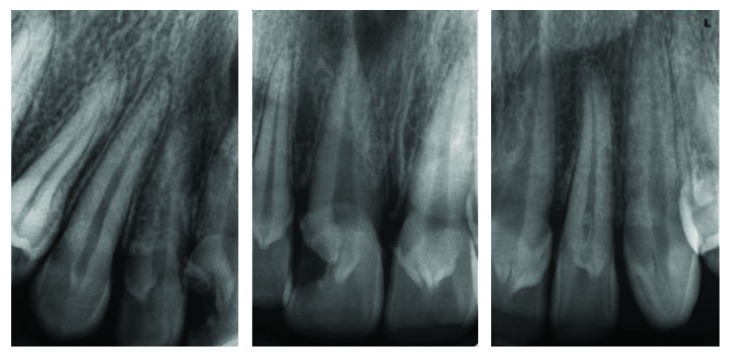
Case 2: periapical radiograph shows radiographic appearance of talon cusp.

Radiographic appearance of talon cusps has been shown in Figure 8.

**Figure 9:**
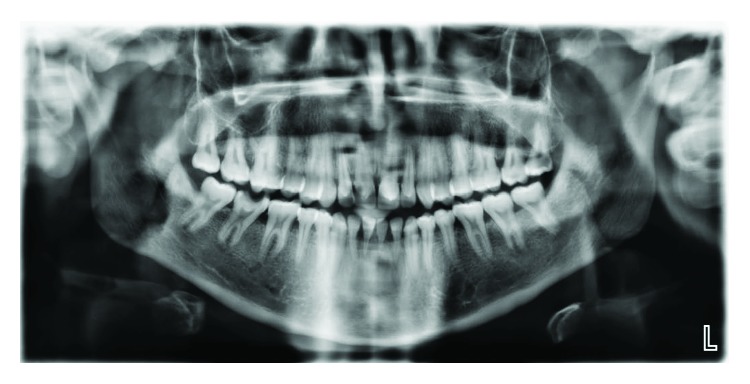
Case 2: dental panoramic tomography (DPT) shows taurodontism in molars.

Talon cusp in this patient is also associated with taurodontism in posterior teeth (Figure 9).

### 2.3. Case 3

**Figure 10:**
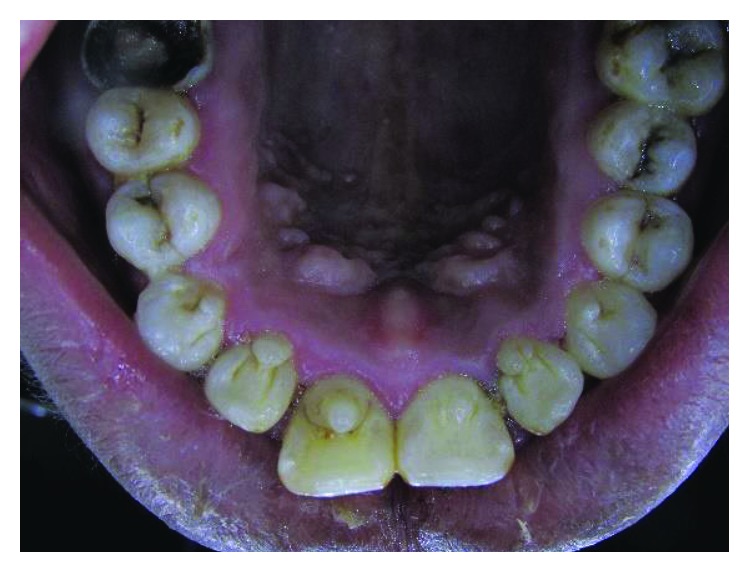
Case 3: palatal view shows talon cusps in all upper anterior teeth.

**Figure 11:**
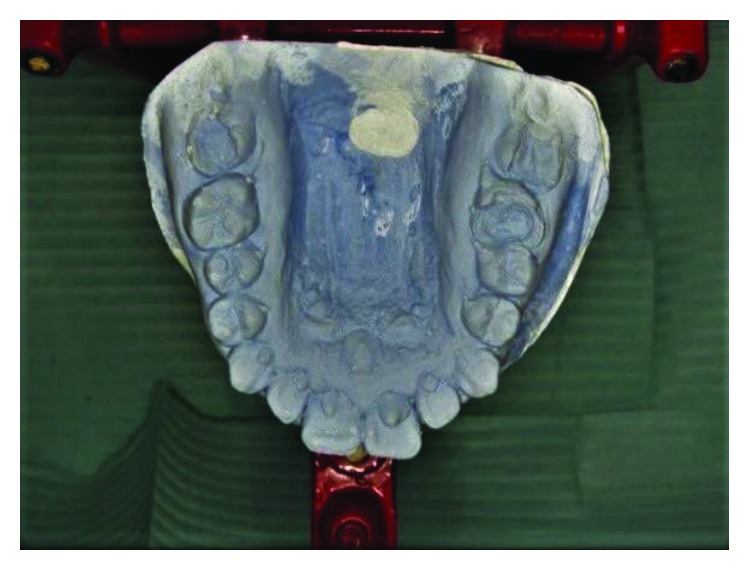
Case 3: talon cusps in all upper anterior teeth (study cast).

**Figure 12:**
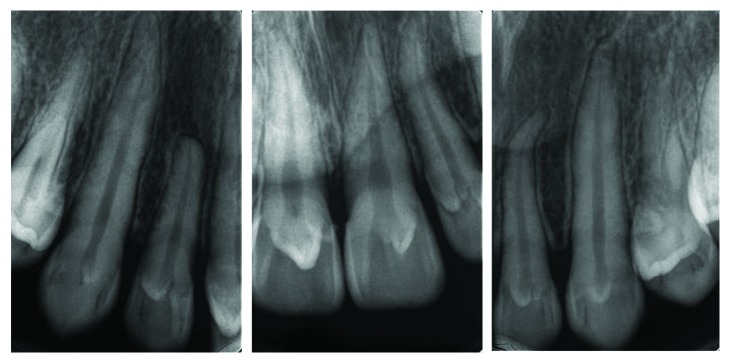
Case 3: periapical radiograph shows radiographic appearance of talon cusps.

A 19-year-old male, the second brother of case 1, has also presented with talon cusps in the palatal aspect of all upper anterior teeth (Figures 10–12).

Dimensions and classification of the tubercles according to Hattab et al. [2] have been shown in Table 1.

The tubercle on the right central incisor is detached from the tooth surface and separated with a deep groove, while in the left central incisor, the tubercle is merged with the tooth surface. The tubercles on lateral incisors are separated with shallow grooves, so are the canines (Figures 10 and 11).

**Figure 13:**
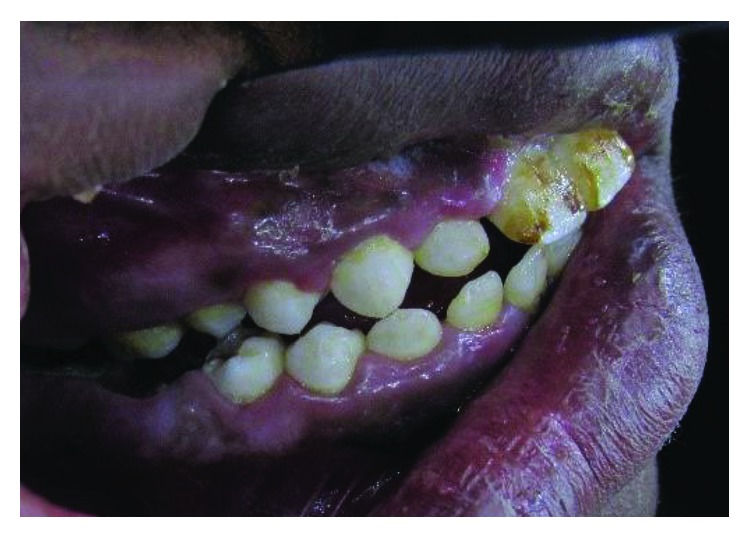
Case 3: small size of teeth which may be due to gingival enlargement or incomplete eruption (right side view).

**Figure 14:**
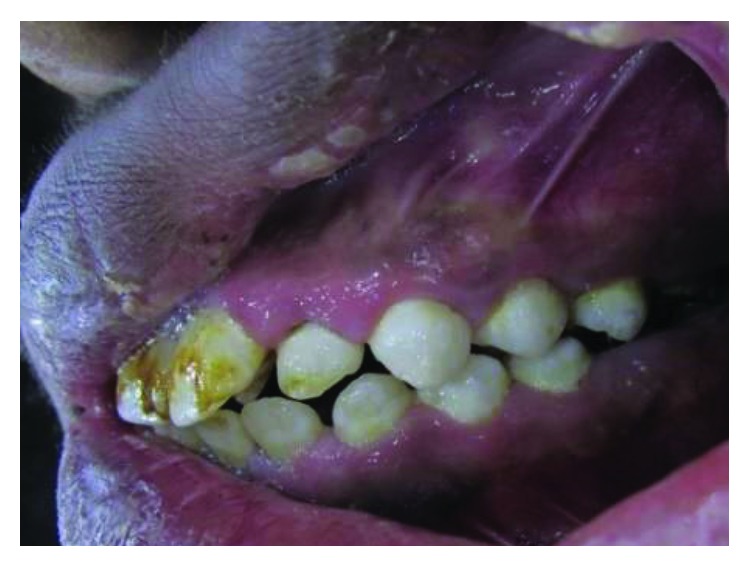
Case 3: small size of teeth which may be due to gingival enlargement or incomplete eruption (left side view).

**Figure 15:**
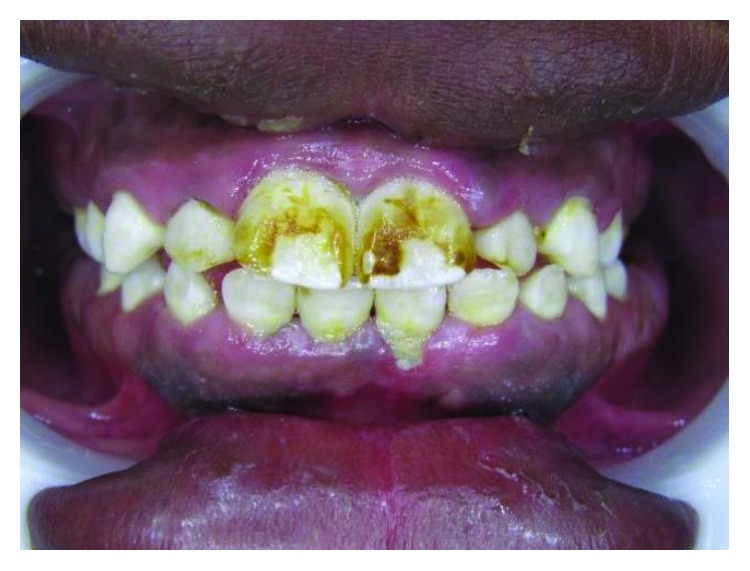
Case 3: small size of teeth which may be due to gingival enlargement or incomplete eruption (labial view).

Clinically, the teeth look generally smaller than normal size, which may be due to gingival enlargement or incomplete eruption. This can only be discovered after gingival treatment and oral hygiene improvement (Figures 13–15).

**Figure 16:**
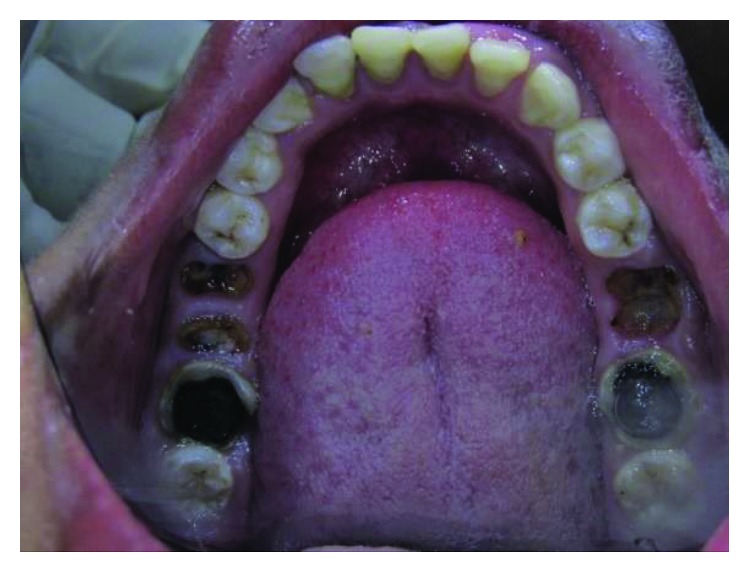
Case 3: shovel-shaped mandibular canines.

On clinical examination, shovel-shape appearance has been noticed on both mandibular canines (Figure 16) and both maxillary lateral incisors (Figure 10).

**Figure 17:**
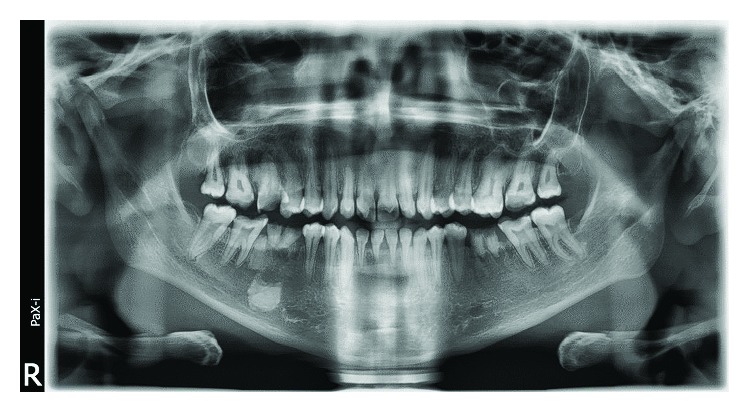
Case 3: dental panoramic tomography (DPT) shows taurodontism in molars.

Taurodontism in molars was shown in dental panoramic tomography (DPT) (Figure 17).

### 2.4. Case 4

**Figure 18:**
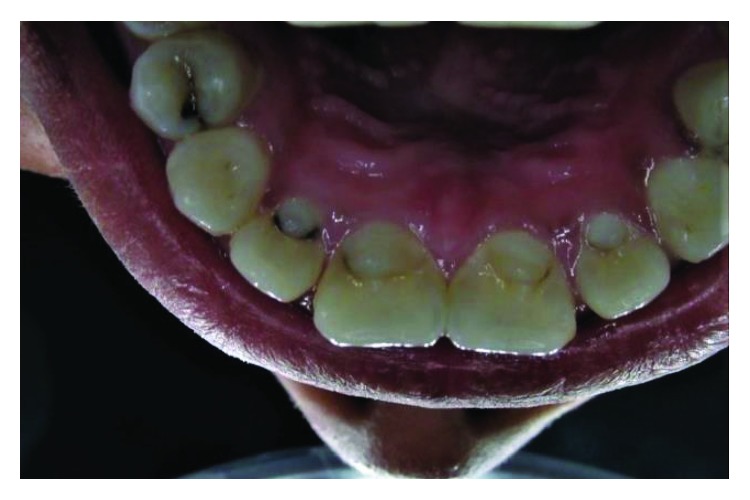
Case 4: talon cusps in all upper anterior teeth.

A 15-year-old female, the youngest sister of case 1, has also presented with talon cusps in all upper anterior teeth. All taloned teeth have responded normally to the sensibility test. On both central and lateral incisors, there are grooves separating talon cusps from the palatal surfaces of the teeth. The grooves are carious except that on the left central incisor (Figure 18).

**Figure 19:**
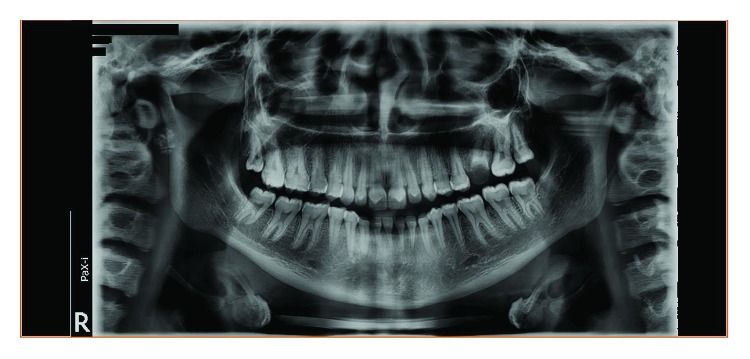
Case 4: dental panoramic tomography (DPT) shows taurodontism in molars.

As in the previous three cases, taurodontism in molars has appeared in DPT (Figure 19).

**Figure 20:**
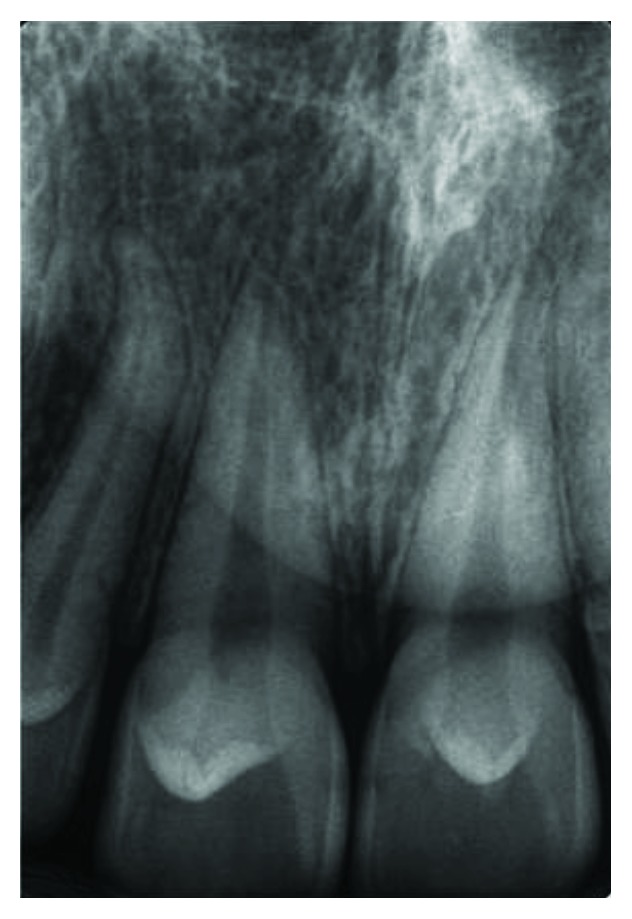
Case 4: periapical view for central incisors.

The usual V-shape radiographic appearance of talon cusps is clearly shown in the periapical view and DPT (Figures 19 and 20).

Table 1 shows dimensions of talon cusps of this case and its classification according to Hattab et al. [2].

**Figure 21:**
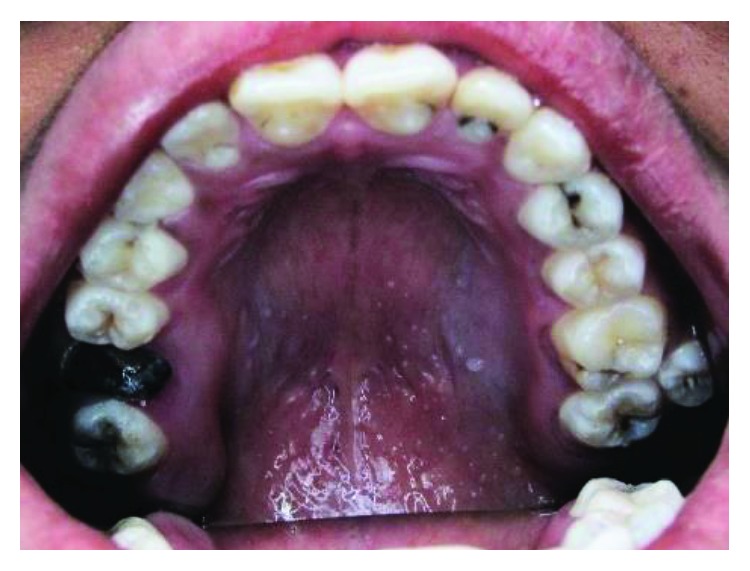
Case 4: supernumerary tooth (paramolar) associated with talon cusps (occlusal view).

Clinical examination has revealed a supernumerary tooth (paramolar) on the right upper posterior quadrant (Figure 21).

**Figure 22:**
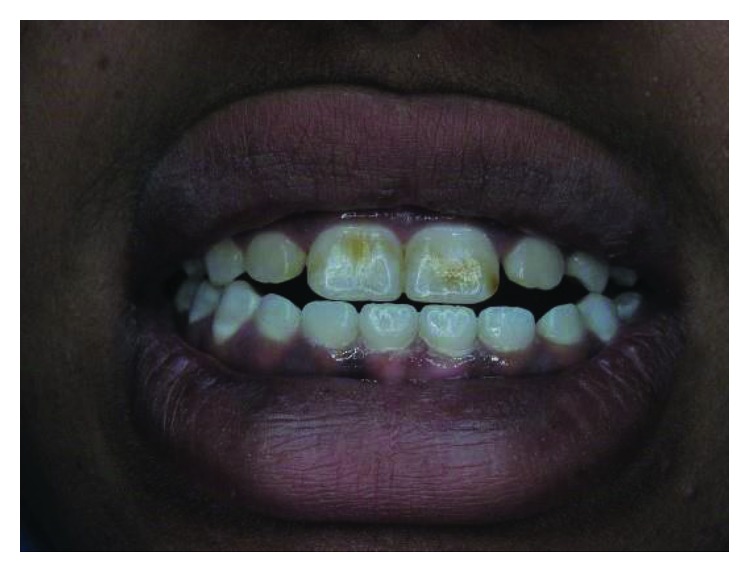
Case 4: unusual small teeth due to incomplete eruption (labial view).

General examination of the teeth has revealed abnormally small teeth which may be due to incomplete eruption of teeth (Figure 22).

### 2.5. Case 5

**Figure 23:**
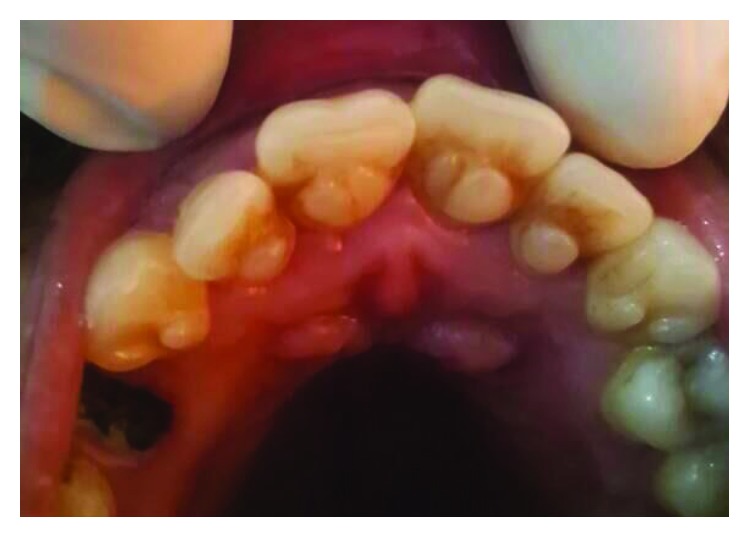
Case 5: talon cusps in all upper anterior teeth.

A 27-year-old male, the oldest brother of case 1, like his siblings, has also talon cusps in all anterior maxillary teeth. Unfortunately, this patient works in a far rural area. He could not come for dental examination. Photos for this case have been sent by a medical officer who works there (Figure 23).

**Figure 24:**
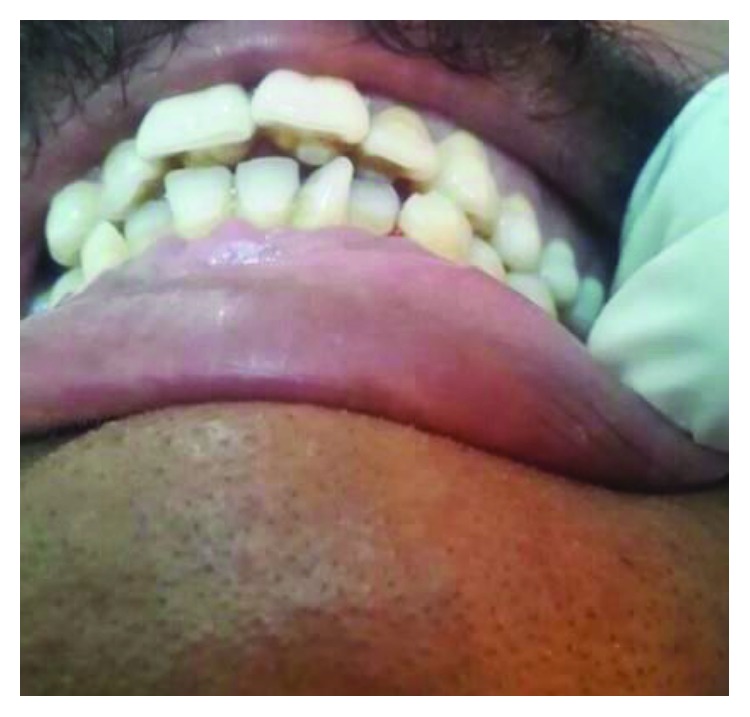
Case 5: occlusal interference by talon cusps. Proclination of upper anterior teeth occurs as a result of this interference.

Talon cusps interfere with the occlusion of anterior teeth. This interference has resulted in a slight malalignment on anterior teeth (Figure 24).

Dental variations associated with talon cusp anomaly in this report have been summarized in Table 2.

## 3. Discussion

Talon cusp is an uncommon condition which affects primary and permanent teeth. Literature is not rich with data of talon cusp per se. Most of the papers concerning talon cusp have entitled and focused on dens evaginatus, as previous literature considers them as one entity.

Up to 2014, the total number of articles published on “talon cusp” is about 170 articles that described 407 teeth with talon cusp. Of the total 407 taloned teeth, 375 talon cusps were located on the palatal/lingual surface while 32 cusps were located on the facial surface. The same reviews reveal that 309 out of 407 taloned teeth were permanent whereas 98 were primary teeth [12].

The prevalence of talon cusp varies according to populations. Sedano et al. in a survey done in Mexico to determine dental anomalies in children found that this condition was present in only 0.06% [20]. Also, a smaller survey done on 536 patients in Malaysia showed that 5.2% of children had talon cusp in one of their incisors [21].

According to Hattab's review, the prevalence of talon cusp ranges from 0.06% to 7.7% and there is a racial variation for distribution of this anomaly. It occurs with a higher incidence in the Arab population than in Caucasians and Blacks [12]. This report has added to literature 30 taloned teeth in five cases that belong to the Arab population (Sudanese). Although prevalence of talon cusp in the Sudanese population has not been studied yet, according to the author's clinical experience, it is not very uncommon.

Literature reported familial evidence of talon cusp: the anomaly has been described affecting two siblings [16, 22], two sets of female twins [23], two family members [24], and three siblings [25]. Based on this data, the literature supports the hereditary character of talon cusp [13]. This report of five siblings with talon cusps adds to literature a firm evidence of a genetic background for this anomaly.

It has been noticed that sometimes talon cusp affected patients who had consanguineous parents [2, 24]. Hattab et al. mentioned that 8 out of 13 cases with talon cusp had first-cousin parents [1, 2]. This report is compatible with which had been mentioned in the literature, as parents of the five siblings are cousins.

Mader suggested that talon cusp may be associated with other somatic and odontogenic anomalies like peg-shaped lateral incisors and impacted mesiodens and canine [26]. Natkin et al. reported a case of talon cusp associated with complex odontoma and impaction [6]. Davies and Brook found that talon cusp is associated with supernumerary teeth, megadont, and dens evaginatus [27]. Hattab added a number of dental variations that may be associated with talon cusp. It includes ectopic canine, hypodontia, double teeth, dens invaginatus, shovel-shaped incisors, bifid cingula, and exaggerated Carabelli cusp [12]. Taurodontism has never been mentioned in literature to be associated with talon cusp. The present report is the first one that has mentioned taurodontism as one of the dental anomalies that may associate talon cusp.

The treatment of talon cusp requires careful clinical and radiographic judgment. Each case should be assessed separately. Size of talon cusp plays an important role in the treatment plan. A small cusp which is asymptomatic needs no treatment while a large prominent cusp associated with functional and esthetic problems like case 1 requires definitive treatment.

The groove that separates talon cusp from the tooth surface requires especial considerations. If talon cusp blends with the tooth surface with no definite groove that delineates it like in the left central incisor in case 3, no preventive measure should be taken. However, a preventive measure should be taken if the groove is deep but noncarious: it should be cleaned from debris and plaque and prophylactically sealed with a fissure sealant. In case of carious grooves, then the lesion should be eradicated and the cavity filled with glass ionomer cement [2].

The anomalous cusp should be reduced in case of occlusal interference [2]. The amount of pulp extension within talon cusp is one of the challenges that may face the clinician when cusp reduction is required. Grinding of the palatal projection must be performed, with the possibility of exposure of the dentin-pulp complex, and consequently, pulp necrosis may be expected [28]. However, many grinding trials to remove occlusal interference of talon cusp did not end up with pulp exposure. Pitts and Hall reduce a talon cusp by 3 mm in one visit without pulp exposure [29]. Hattab et al. suggested to treat the ground surface with fluoride varnish (Duraphat) as a desensitizing agent to enhance reparative dentin formation [30]. One to 1.5 mm has been ground from six talon cusps in one visit also without exposing the pulp [2]. Dankner et al. have performed selective grinding for talon cusp in the lower mandibular incisor through sessions which continue for 12 months. Each grinding session was followed by application of Duraphat sodium fluoride gel. After 10 years of follow-up period, the tooth remains asymptomatic and vital [31]. Hattab and Hazza'A have tried to complete grinding of the cusp on two sessions at 6- to 8-week intervals without exposing the pulp [32].

Superimposition of talon cusp over the tooth crown makes it difficult to use ordinary radiographic evaluation like periapical radiograph or dental panoramic tomography to trace the pulp extension within the cusp. Accordingly, the author has suggested computed tomography before commencing in treatment of some cases of large separated talon cusp. Evaluation of pulp extension within the tubercle will be more accurate this way.

**Figure 25:**
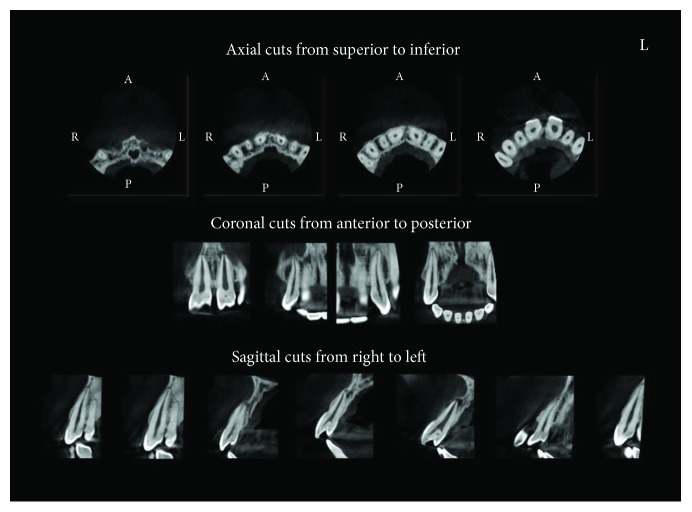
Cone beam computed tomography (CBCT) for the anterior segment of case 1.

The treatment plan for case 1 was challenging due to the risk of pulp exposure following grinding of the large tubercles in the central incisors. Risk of exposing the pulp is critical for the right central incisor rather than the left as the left central incisor has already been necrotic. Radiographic evaluation for case 1 has been done using cone beam computed tomography (CBCT) (Figure 25).

The four accessible siblings have been contacted and invited for dental examination. They have poor oral hygiene, gingivitis, and multiple carious teeth. Free dental treatment has been offered in the university clinic. However, they live in remote rural areas making it difficult to follow up with treatment.

## 4. Conclusion

Talon cusp is a significant anomaly with variant clinical impacts. Early discovery of this anomaly can diminish or prevent the clinical problems which are associated with it. This report is a strong evidence of a genetic background of talon cusp. So, if a patient present with talon cusp, it is advisable to examine his/her siblings to provide an early preventive measure in case they have the same anomaly.

## Figures and Tables

**Table 1:** Comparison between measurements of talon cusps of the five cases and Hattab et al.'s classification for each tubercle.

Tooth type	Case number				
	Case 1	Case 2	Case 3	Case 4	Case 5
Right central incisor 11	IG = 6 mm MD = 6 mm Type 1 (talon)	IG = 5 mm MD = ?? Type 1 (talon)	IG = 5 mm MD = 4 mm Type 1 (talon)	IG = 4.5 mm MD = 4.5 mm Type 2 (semitalon)	No clinical examination (out of reach)

Right lateral incisor 12	IG = 4.5 mm MD = 4 mm Type 2 (semitalon)	IG = 4 mm MD = 3 mm Type 2 (semitalon)	IG = 3 mm MD = 3 mm Type 2 (semitalon)	IG = 3 mm MD = 3 mm Type 2 (semitalon)	No clinical examination (out of reach)

Right canine 13	IG = 4 mm MD = 3 mm Type 2 (semitalon)	Enlarged bifid cingulum Type 3 (trace talon)	IG = 3 mm MD = 2.5 mm Type 2 (semitalon)	Enlarged cingulum Type 3 (trace talon)	No clinical examination (out of reach)

Left central incisor 21	IG = 7 mm MD = 7 mm Type 1 (talon)	IG = 5.5 mm MD = 5 mm Type 1 (talon)	IG = 5 mm MD = 5 mm Type 1 (talon)	IG = 4.5 mm MD = 4 mm Type 2 (semitalon)	No clinical examination (out of reach)

Left lateral incisor 22	IG = 4 mm MD = 4 mm Type 2 (semitalon)	IG = 4 mm MD = 3 mm Type 2 (semitalon)	IG = 4 mm MD = 3.5 mm Type 2 (semitalon)	IG = 3 mm MD = 3 mm Type 2 (semitalon)	No clinical examination (out of reach)

Left canine 23	IG = 4 mm MD = 4 mm Type 2 (semitalon)	Enlarged bifid cingulum Type 3 (trace talon)	IG = 3 mm MD = 2.5 mm Type 2 (semitalon)	IG = 3 mm MD = 3 mm Type 2 (semitalon)	No clinical examination (out of reach)

MD: the mesiodistal dimension of the tubercle (in millimeters); IG: the incisogingival dimension of the tubercle (in millimeters); ??: the dimension could not be measured due to caries destruction.

**Table 2:** Summary of dental anomalies associated with talon cusp in the report.

Case number	Associated dental anomaly		
Case 1	Taurodontism	Dens invaginatus	

Case 2	Taurodontism	Bifid cingula	

Case 3	Taurodontism	Shovel-shaped anterior teeth	Small teeth size (questionable if it is due to incomplete eruption or inflammatory gingival enlargement)

Case 4	Taurodontism	Supernumerary tooth (paramolar)	Small teeth size (questionable if it is due to incomplete eruption or inflammatory gingival enlargement)

Case 5	Clinical examination was not done		

## Abbreviations

MD: Mesiodistally
IG: Incisogingivally
DPT: Dental panoramic tomography.
